# Defective small intestinal anion secretion, dipeptide absorption, and intestinal failure in suckling NBCe1-deficient mice

**DOI:** 10.1007/s00424-016-1836-3

**Published:** 2016-05-26

**Authors:** Qin Yu, Xuemei Liu, Yongjian Liu, Brigitte Riederer, Taolang Li, De-An Tian, Biguang Tuo, Gary Shull, Ursula Seidler

**Affiliations:** Department of Gastroenterology, Hannover Medical School, Carl-Neuberg-Str. 1, 30625 Hannover, Germany; Department of Gastroenterology, Tongji Hospital, Huazhong University of Science & Technology, Wuhan, People’s Republic of China; Department of Gastroenterology, Zunyi Medical College, Zunyi, China; Department of Gastrointestinal Surgery, Zunyi Medical College, Zunyi, China; Department of of Molecular Genetics, University of Cincinnati, Cincinnati, OH USA

**Keywords:** Bicarbonate, pH_i_ regulation, Anion exchange, PEPT-1, Sodium–bicarbonate cotransporter

## Abstract

The electrogenic Na^+^HCO_3_^−^ cotransporter NBCe1 (Slc4a4) is strongly expressed in the basolateral enterocyte membrane in a villous/surface predominant fashion. In order to better understand its physiological function in the intestine, isolated mucosae in miniaturized Ussing chambers and microdissected intestinal villi or crypts loaded with the fluorescent pH-indicator BCECF were studied from the duodenum, jejunum, and colon of 14- to 17-days-old *slc4a4*-deficient (KO) and WT mice. NBCe1 was active in the basal state in all intestinal segments under study, most likely to compensate for acid loads imposed upon the enterocytes. Upregulation of other basolateral base uptake mechanism occurs, but in a segment-specific fashion. Loss of NBCe1 resulted in severely impaired Cl^−^ and fluid secretory response, but not HCO_3_^−^ secretory response to agonist stimulation. In addition, NBCe1 was found to be active during transport processes that load the surface enterocytes with acid, such as Slc26a3 (DRA)-mediated luminal Cl^−^/HCO_3_^−^ exchange or PEPT1-mediated H^+^/dipeptide uptake. Possibly because of the high energy demand for hyperventilation in conjunction with the fluid secretory and nutrient absorptive defects and the relative scarcity of compensatory mechanisms, NBCe1-deficient mice developed progressive jejunal failure, worsening of metabolic acidosis, and death in the third week of life. Our data suggest that the electrogenic influx of base via NBCe1 maintains enterocyte anion homeostasis and pH_i_ control. Its loss impairs small intestinal Cl^−^ and fluid secretion as well as the neutralization of acid loads imposed on the enterocytes during nutrient and electrolyte absorption.

**Electronic supplementary material:**

The online version of this article (doi:10.1007/s00424-016-1836-3) contains supplementary material, which is available to authorized users.

## Introduction

Intestinal HCO_3_^−^ transport is of paramount importance for intestinal absorptive, secretory, and barrier function [2, 7, 8, 25, 33, 35]. Mutations in the two major HCO_3_^−^ exit pathways into the lumen, namely CFTR and DRA (Slc26a3), cause severe intestinal disease in humans [9, 15, 27], and the corresponding gene knockout is lethal for mice, pigs, and ferrets due to their intestinal phenotypes [20, 26, 32]. Surprisingly, however, no intestinal phenotypes have been described in humans associated with genetic alterations of basolateral HCO_3_^−^ transport proteins. Is this due to redundancy? Do we sufficiently understand the functions of basolateral acid/base transporters in the intestine?

Current dogma envisions intestinal HCO_3_^−^ uptake through the basolateral membrane to be mediated either by a Na^+^HCO_3_^−^ cotransporter or by CO_2_ hydration followed by proton extrusion via basolateral Na^+^/H^+^ exchangers [5, 7, 34]. Experimental evidence for this concept has been provided in the rabbit duodenum, and it has been shown that both pathways need to be inhibited to significantly reduce the HCO_3_^−^ secretory response to agonists [21].

Several NBC isoforms are expressed in the intestinal tract, namely the electroneutral NBCn1, and the electrogenic NBCe1 and NBCe2 isoforms [7, 10, 14]. The electrogenic NBCe1 is expressed in several splice variants with different N- or C-termini, resulting in differential organ expression and stoichiometry [1]. The electrogenic Na^+^HCO_3_^−^ cotransporter NBCe1B (Slc4a4) is expressed throughout the gastrointestinal epithelium [23]. In the pancreatic ducts, the NBCe1B isoform serves to import HCO_3_^−^ during agonist-stimulated ductal bicarbonate secretion, and the orchestration between the basolateral activation of NBCe1 and the apical CFTR occurs via the IRBIT protein, which binds to and regulates both transporters [28]. Similarities between the ion transport mechanisms in pancreatic ductal and intestinal epithelium suggested that the intestinal NBCe1B is a transporter important for intestinal HCO_3_^−^ secretion [5, 7, 33, 35]. NBCe1 KO mice are acidotic and die within 3 weeks of life, but have small intestinal impactions at the time of death. Gawenis et al. [16] found that proximal colonic mucosal agonist-induced I_sc_ was reduced compared to WT mucosa when only HCO_3_^−^ anion was present in the luminal and serosal perfusate and when CO_2_ hydration was simultaneously inhibited by acetazolamide, suggesting that under these ionic conditions, NBCe1 is involved in basolateral HCO_3_^−^ uptake during electrogenic HCO_3_^−^ secretion in the murine proximal colon. Because of the early death and stunted growth of these mice, the intestinal tract is tiny at the time of experimentation and thus other parts of the intestine were not investigated. The electroneutral NBCn1 isoform has a particularly strong expression in the duodenum [14] and is essential for agonist-induced and more so for acid-induced bicarbonate secretion [10, 38]. The electrogenic NBCe2 is expressed at low levels in the adult murine small intestine [10], and heterologous expression studies in mammalian epithelial cells suggest a 3:1 coupling for HCO_3_^−^ and Na^+^, and therefore an outward flux of HCO_3_^−^ under physiological conditions [reviewed in 1].

Recent immunohistochemical studies by Jakab et al. [23] elucidated the expression of NBCe1 along the proximal to distal as well as crypt to villus axes of the rat intestinal tract. NBCe1 expression was found predominantly in small intestinal villous and colonic surface cells. Except in the duodenum, these cells are not those where CFTR is strongly expressed, which is crypt-predominant in most parts of the intestinal tract [3, 23]. On the other hand, it is well documented that for the cAMP-dependent HCO_3_^−^ secretory response, CFTR expression is essential. While this does not rule out that in certain cells along the crypt–villus axis, CFTR and NBCe1 are coexpressed, it does suggest that intestinal NBCe1 may also be involved in other biological functions than electrogenic anion secretion. The present work was therefore carried out to study pH_i_ control, electrophysiology, anion secretion, and nutrient absorption, as well as the adaptive response of the different murine intestinal segments to the lack of NBCe1 expression.

## Material and methods

### Animals

The Slc4a4-gene-deleted mouse strain was originally generated by the group of Gary Shull and its major characteristics have been described before [16]. Experiments were performed on *Slc4a*4^+/+^ and ^−/−^ littermates, congenic on the 129/SVJ background, at 1z4-17 days of age, or the age indicated in the respective figure. *Slc4a7*^−/−^ (NBCn1 KO) and WT mice, generated in the laboratory of Christian Aalkjaer, were raised and genotyped as described previously [10, 38]. The genotypes of the mice were verified by PCR. Mice were killed by cervical dislocation after light isoflurane anesthesia, the intestine was excised and immediately placed in ice-cold oxygenated Ringer’s solution, pH 7.4 and washed before further processing. All studies were approved by the committee on investigations involving animals, Hannover Medical School, and an independent committee assembled by the local authorities.

### Ussing chamber experiments

Ussing chamber experiments destined to assess HCO_3_^−^ secretory rate were performed in the open-circuit mode and alkaline output into the lumen was continuously assessed by pH-stat titration. The potential difference (PD) and *R*_t_ were measured and *I*_sc_ was calculated as described previously [29]. For the experiments to examine glycilsarcosin [(GlySar), a nonhydrolysable dipeptide transported by PEPT1]-induced *I*_sc_, voltage clamp conditions with bilaterally identical solutions (except for a lack of glucose in the luminal bath) were used as described [11]. To prevent osmotic gradients during luminal GlySar application, an identical molar concentration of mannitol was added to the basolateral side. For Cl^−^-free experiments, Na^+^-gluconate replaced NaCl in the luminal bath. Solution compositions are given in Electronic supplementary material (ESM) Table 1. Colonic tissue was studied in the presence of 10^−5^ amiloride in the luminal bath to inhibit apically expressed ENaC, unless otherwise stated.

### Isolation of colonic crypts

Intact colonic crypts were isolated from inverted proximal and mid-distal colonic segments separately by a Ca^2+^ chelation method described previously [4, 12], except for the use of 1 mM EDTA instead of 5 mM in the Ca^2+^-free chelation solution, and the use of the ice-cold bicarbonate buffered solution A with 1 % bovine serum albumin (BSA) for preparing the colon and harvesting the colonic crypts. Proximal colon was the first 2 cm from the cecocolonic junction, and mid-distal colon was from ~3 cm after the cecocolonic curvature, ending about 1 cm away from the anus.

### Preparation of isolated duodenal and jejunal villi

Isolation of intact duodenal and jejunal villi for fluorometry were performed as previously described [10, 11], except for the use of an O_2_/CO_2_-gassed, bicarbonate buffered solution (buffer A in ESM Table 2)

### Assessment of intracellular pH (pH_i_) and base influx rates

Steady-state pH_i_ was assessed by measuring BCECF fluorescence in the different regions of the colonic crypt and small intestine villi for 20 min during stable conditions, then performing a calibration in a very narrow pH range (in which the steady-state pH_i_ is expected), as described by Hegyi et al. [18]. Base influx rate was measured by multiplying the Na^+^-dependent, Hoe642 (50 μM inhibits NHE1 and NHE2 [6]) and S1611 (20 μM inhibits NHE3)-independent pH_i_-recovery from an intracellular acid load in the initial linear recovery phase with the total buffering capacity (intrinsic and CO_2_/HCO_3_^−^ mediated) at the mean pH_i_ during this recovery phase, as previously described [6. 11, 12, 38]. The intrinsic buffer capacity was determined as described in [4, 11]. The detailed buffer composition is given in ESM Table 2.

### mRNA expression of ion transport proteins in the gastrointestinal tract

mRNA expression levels in scraped mucosa of different parts of intestine were performed using a quantitative real-time PCR protocol and the primer pairs were used as described before [10]. The mRNA expression of other acid/base transporters was assessed by qPCR in relation to the geometric mean of a set of marker genes (epithelium-specific, villus or crypt/basal villus predominant, total RNA), because we know from previous experiments that no single control gene is homogenously expressed along the crypt–villus axis and in the different segments.

### Histology

The different intestinal segments were excised, fixed with 4 % paraformaldhyde and embedded in paraffin. Tissue sections (2 μm) were prepared, deparaffinized, and stained with hematoxylin and eosin by standard protocols. No visible differences were apparent on gross morphological examination of the different intestinal segments under study (ESM Fig. 1).

### Statistics

Data are presented as means ± SEM. *n* indicates the number of pairs (WT and KO), or, if a range is given, the number of WT and KO mice. The Mann–Whitney rank-sum test, student’s *t* test or, if appropriate, the ANOVA for multiple comparisons were used for statistics, and values of *P <* 0.05 were considered significant (*P < 0.05, **P < 0.01, ***P < 0.001).

## Results

### Steady-state pH_i_ and base influx rates in duodenal, jejunal, proximal and distal colonic mucosa

Steady-state pH_i_ was not different between NBCe1 KO and WT small intestinal enterocytes either in the presence or absence of Cl^−^ in the perfusate. In NBCe1 KO proximal surface colonocytes, a significantly reduced steady-state pH_i_ was observed compared to WT (Table 1). NBCe1 KO colonic crypt cells and the NBCn1 WT colonic surface and crypts cells did not display significant differences in steady-state pH_i_ (data not shown).

**Table 1:** Steady-state pHi in the duodenal, jejunal, proximal colonic mucosa of NBCe1 WT and KO mice

Mice	Cl^−^-containing perfusate		Cl^−^-free perfusate (inhibits AEs and NCBEs)		
	Duodenal villi	Jejunal villi	Duodenal villi	Jejunal villi	Prox. surface colonocytes
NBCe1^+/+^	7.24 ± 0.06	7.16 ± 0.03	7.46 ± 0.07	7.46 ± 0.08	7.78 ± 0.05
NBCe1^−/−^	7.28 ± 0.05	7.16 ± 0.04	7.47 ± 0.08	7.43 ± 0.05	7.66 ± 0.04^*^

*P < 0.05, *n* = 7–10

**Fig. 1:**
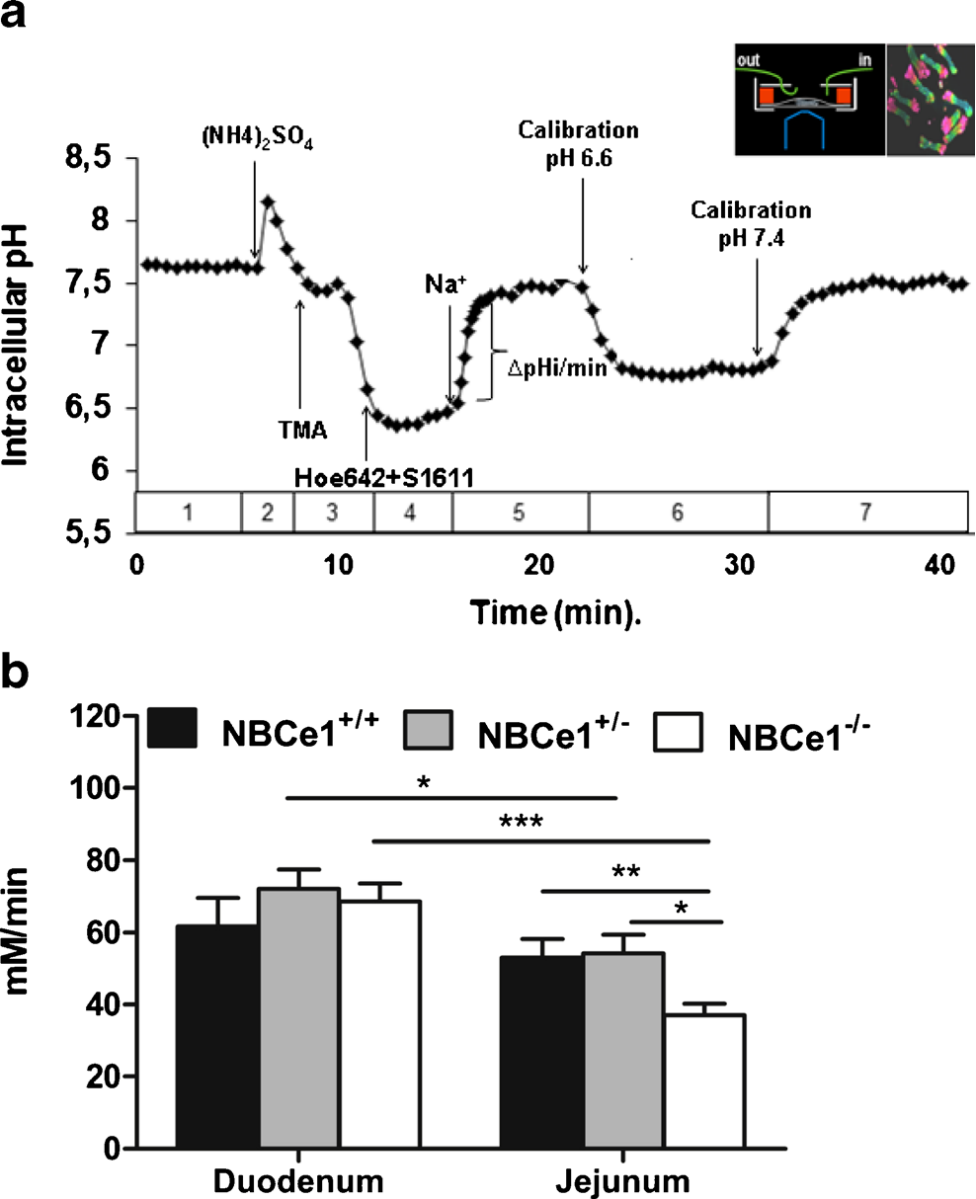
NBC-mediated base influx rates in duodenal and jejunal villous enterocytes. **a** Time course of an ammonium-prepulse-acidification followed by pH_i_-recovery experiment. **b** Base influx rates after intracellular acidification into duodenal and jejunal enterocytes in the absence of Cl^−^ and presence of 50 μM Hoe642 and 20 μM S1611 to inhibit NHE1-3. A significant reduction in base influx rates was observed in jejunal, but not duodenal NBCe1 KO enterocytes. *P < 0.05, *n* = 9–10

**Fig. 2:**
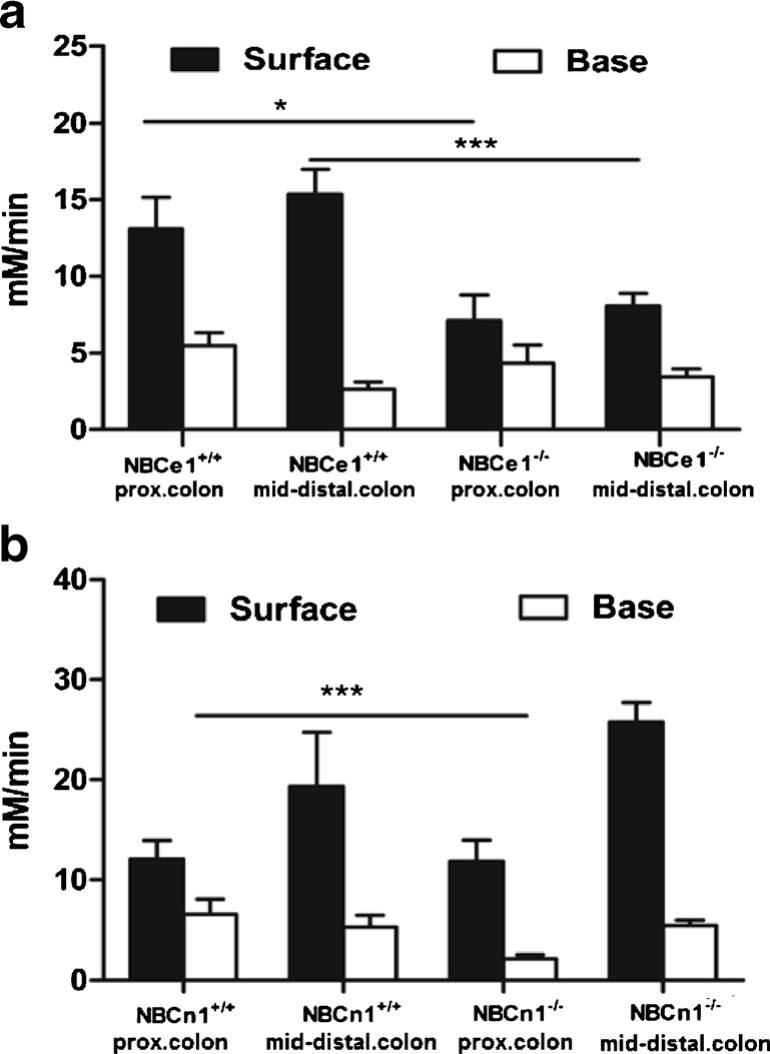
NBC-mediated base influx rates in colonic surface and cryptal base cells. **a** NBCe1 KO surface but not cryptal base colonocytes from the proximal as well as distal colon displayed significantly lower base influx rates than WT cells. **b** In contrast, a significant difference was observed in the base influx rates into NBCn1 KO and WT proximal colonic crypt cells. In all mouse genotypes, the surface colonocytes had both a higher steady-state pH than the cryptal base cells (see results), as well as higher base influx rates. *n* = 5–6, *P < 0.05

The assessment of Na^+^ and HCO_3_^−^-dependent pH_i_ recovery in the presence of Hoe642 to inhibit NHE isoforms, and in the absence of Cl^−^ to inhibit Cl^−^-dependent Na^+^HCO_3_^−^ exchangers, yielded significantly lower base influx rates in jejunal villous NBCe1 KO compared to WT enterocytes, and in surface (cryptal mouth) NBCe1 KO compared to WT colonocytes from the proximal as well as the distal colon (Figs. 1a, b and 2a). In duodenal villi and in the colonocytes at the base of the crypts, no significant differences in base influx rates between NBCe1 KO and WT enterocytes were observed. NBCn1-deficient colonocytes had lower base influx rates after an acid load in the basal crypt cells of the proximal but not the distal colon (Fig. 2b).

### Basal and FSK-stimulated HCO_3_^−^ secretion in the different segments of the NBCe1 KO mouse

**Fig. 3:**
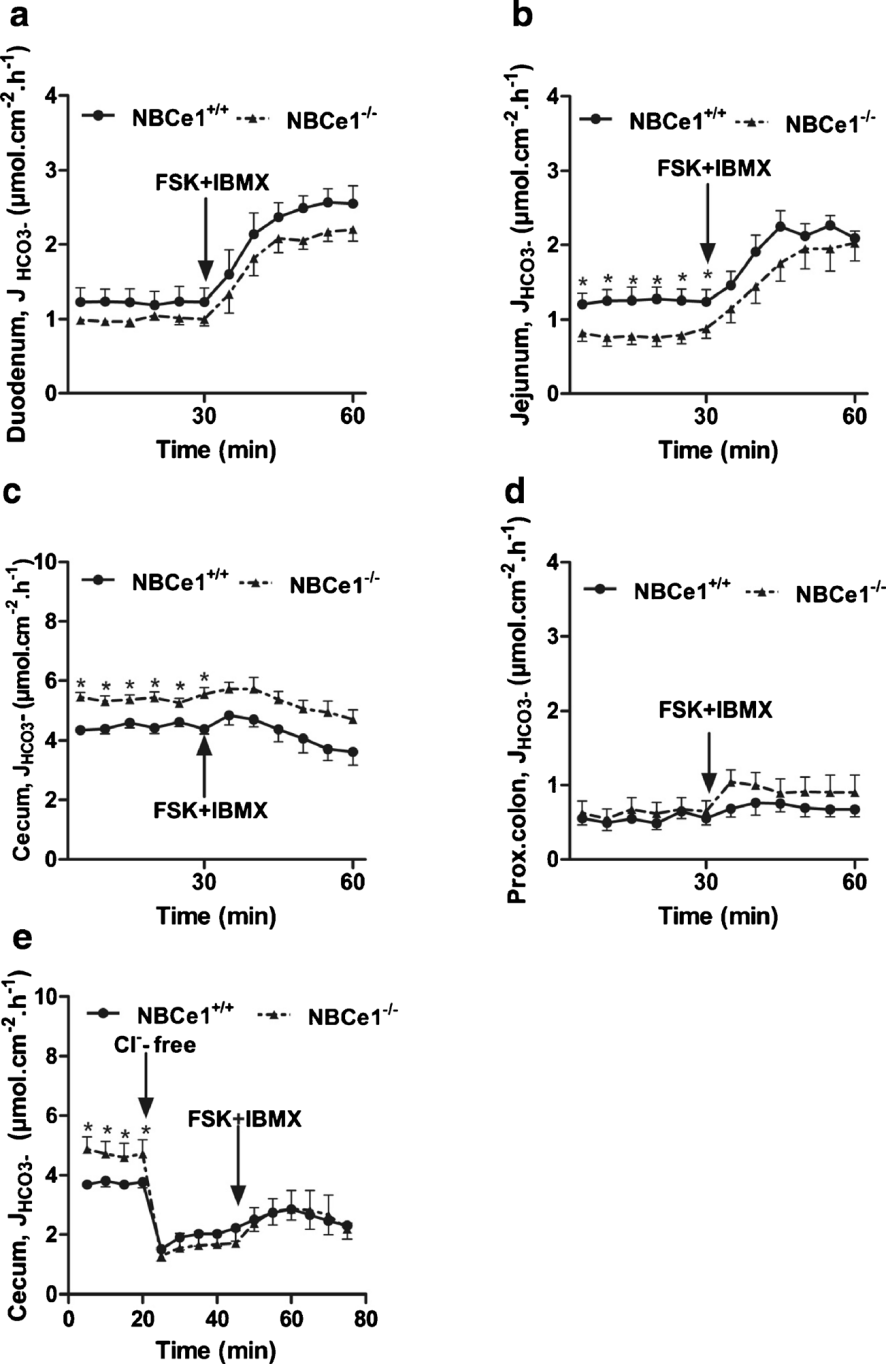
Effect of NBCe1 ablation on basal and FSK-stimulated HCO_3_^−^ secretion. **a**–**d** Time course of luminal alkalinisation rates in duodenum (**a**, *n* = 7), jejunum (**b**, *n* = 8), cecum (**c**, *n* = 6) and proximal colon (**d**, *n* = 7) in NBCe1 KO and WT mucosa. Alkalinization rates were significantly lower in the jejunum of NBCe1 KO mice, and significantly higher in the cecum. The high cecal alkalinisation rates are due to a high expression of DRA and a low expression of NHE3 (as compared to duodenum and jejunum). Removal of Cl- from the luminal bath reduced the alkalinisation rates and abolished the differences between NBCe1 KO and WT (**e**, *n* = 6)

Basal HCO_3_^−^ secretory rate (J_HCO3_^−^) was not significantly different from WT in NBCe1-deficient duodenal mucosa (Fig. 3a), whereas it was significantly lower in jejunal mucosa (Fig. 3b). Basal J_HCO3_^−^ was very high in the cecal mucosa both in WT and, surprisingly, significantly more so in the NBCe1 KO mucosa (Fig. 3c). This high basal J_HCO3_^−^ is most likely due to high DRA (Slc26a3) expression [40, 45]. Consistent with this hypothesis, removal of Cl^−^ from the luminal bath resulted in a dramatic decrease in J_HCO3_^−^, and the residual J_HCO3_^−^ was not different between NBCe1 WT and KO cecal mucosa, indicating that a higher rate of apical Cl^−^/HCO_3_^−^ exchange in NBCe1 KO mice is responsible for the difference (Fig. 3e). In proximal colonic mucosa, where DRA expression is low [24, 40], J_HCO3_^−^ was very low and not different between WT and NBCe1 KO (Fig. 3d).

Surprisingly, the HCO_3_^−^ secretory response (∆*J*_HCO3_^−^) to FSK was not significantly different between WT and NBCe1 KO mucosa (Fig. 3a-d). Since we have previously observed that the CFTR-dependent, FSK-stimulated HCO_3_^−^ secretory response in the murine colon was enhanced by the removal of Cl^−^ from the lumen [46], we also studied the HCO_3_^−^ secretory response to FSK in the cecum in the absence of luminal Cl^−^ (Fig. 3e), but subsequent FSK induced a similar (and small) HCO_3_^−^ secretory response in NBCe1 KO and WT cecum.

### Importance of alternative HCO_3_^−^ uptake/generation pathways for HCO_3_^−^ secretion in the different segments of the NBCe1 KO and WT intestine

**Fig. 4:**
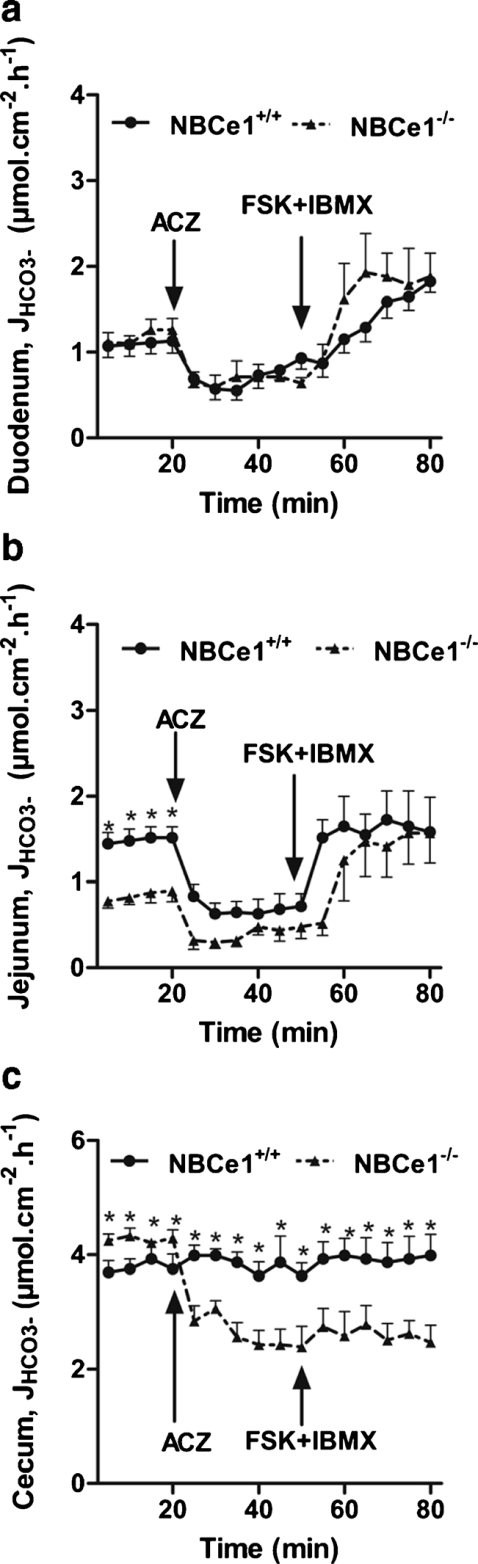
Effect of acetazolamide (ACZ) on HCO_3_^−^ efflux in NBCe1 KO and WT intestinal mucosa. **a**–**c** Blocking the presumed alternative pathway for HCO_3_^−^ production reduced the basal HCO_3_^−^ output in all studied intestinal segments. While the inhibitory effect of ACZ was identical in NBCe1 KO and WT duodenum (**a**, *n* = 7), it was significantly stronger in WT jejunum (*n* = 7), indicating that carbonic anhydrases also augment NBCe1-mediated HCO_3_^−^ supply (**b**). In cecal mucosa (*n* = ,6), the high rates of luminal alkalinisation dramatically decreased after ACZ application in the NBCe1 KO but not the WT, indicating complete compensation of carbonic anhydrase inhibition by NBCe1-mediated HCO_3_^−^ uptake in the latter (and vice versa in the KO)

The addition of acetazolamide to inhibit carbonic–anhydrase facilitated CO_2_ hydration reduced the basal HCO_3_^−^ secretory rate in both the WT and the NBCe1 KO duodenum as well as jejunum (Fig. 4a, b), but the subsequent HCO_3_^−^ secretory response to FSK was not significantly different between the genotypes. This indicates that CO_2_ hydration as a means to generate HCO_3_^−^ for output to the lumen is important in the basal state both in the absence and presence of NBCe1 expression, but that during FSK-stimulated HCO_3_^−^ secretion additional mechanisms for HCO_3_^−^ import operate.

It is of interest that the decrease of HCO_3_^−^ secretory rate in response to acetazolamide was even stronger in the WT compared to the NBCe1 KO jejunum. We assume that this is due to the inhibition by acetazolamide not only of intracellular, but also of membrane-bound carbonic anhydrases, which enhance local HCO_3_^−^ generation and NBCe1-mediated HCO_3_^−^ uptake, as has recently been described for the rat ventricular myocyte [31].

In the cecum, the addition of acetazolamide caused a dramatic decrease in alkalinization rates in the NBCe1 KO cecum, whereas it had no significant effect on the WT epithelium (Fig. 4c). This suggests that the NBCe1 activity is able to fully compensate for the inhibition of CO_2_ generation, as well as vice versa, to maintain the very high luminal alkalinization rates in this segment of the intestine.

### Loss of NBCe1 impairs Cl^−^ and fluid secretion in jejunal mucosa

**Fig. 5:**
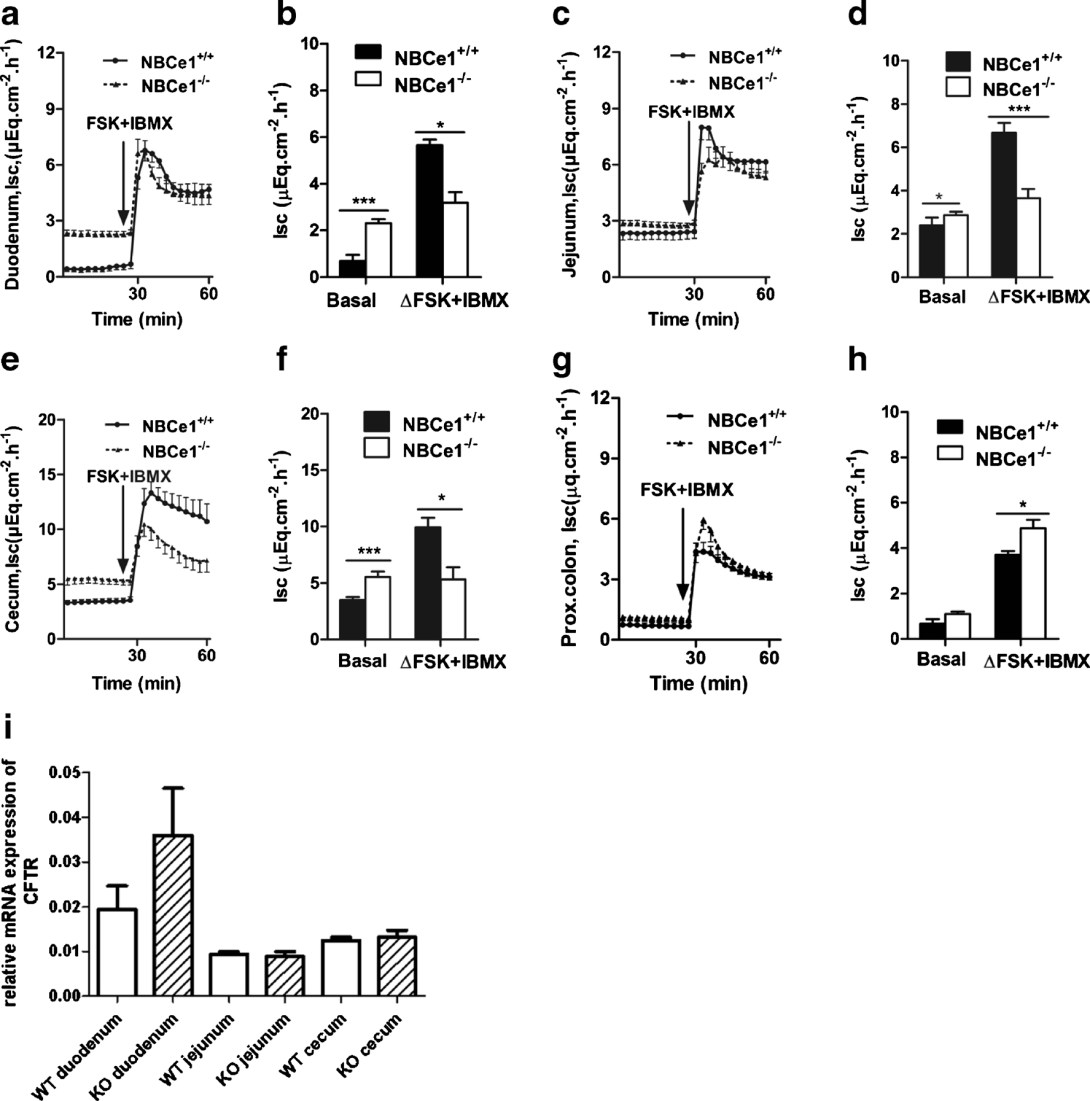
Effect of NBCe1 ablation on short-circuit current and *I*_sc_. **a**–**f** In the small intestine and cecum, but not the proximal colon, basal *I*_sc_ is significantly higher in NBCe1 KO compared to WT mucosa, while the FSK-induced *I*_sc_ response is significantly reduced in jejunal and cecal mucosa (see also Table 2 for PD and *R*_t_ values), but higher in the proximal colon (**g**–**h**). Since an equivalent effect was not found for the *J*_HCO3_^−^ (see Fig. 3), the data indicate an impairment of electrogenic Cl^−^ secretion in the absence of NBCe1 expression in the jejunal and cecal mucosa. CFTR mRNA expression, calculated in relation to the geometrical mean of the expression of villin, actin, RPS9, and cytokeratin 18, was not significantly different between NBCe1 WT and KO intestinal mucosa of the indicated segments. *P < 0.05, duodenum (*n* = 7), jejunum (*n* = 8), cecum (*n* = 6), and proximal colon (*n* = 7)

All studied segments except the proximal colon displayed a significantly higher basal *I*_sc_, as well as PD, in the NBCe1 KO than WT intestine (Fig. 5, Table 2). Furthermore, substances that inhibit NBC (DIDS, SITS, S0859) all resulted in an increase in basal *I*_sc_ in the respective segments of the WT mouse (data not shown).

**Table 2:** FSK-induced *I*_sc_ response and resistance change in the duodenal, jejunal, cecal, and proximal colonic mucosa of NBCe1 WT and KO mice

	PD (mv)		*R*_t_ (Ώ cm^2^)	
	Basal	Peak FSK + IBMX	Basal	Peak FSK + IBMX
Duodenum NBCe1^+/+^	0.34 ± 0.15**	5.78 ± 0.53	24.4 ± 1.60	43 ± 6.81
Duodenum NBCe1^−/−^	1.56 ± 0.09	5.66 ± 0.17	25.8 ± 2.01	44 ± 2.62
Jejunum NBCe1^+/+^	1.28 ± 0.12*	7.18 ± 0.36	23 ± 1.12	40.83 ± 3.25
Jejunum NBCe1^−/−^	1.66 ± 0.04	4.73 ± 0.37*	22.5 ± 1.25	25 ± 1.96^#^
Cecum NBCe1^+/+^	4.14 ± 0.38*	12.76 ± 1.04	43.55 ± 0.89	35.78 ± 1.21
Cecum NBCe1^−/−^	8.35 ± 0.99	13.16 ± 2.19	53.88 ± 6.94	49.2 ± 6.50
Prox. colon NBCe1^+/+^	3.78 ± 0.52	10.38 ± 1.33	68.4 ± 1.80	89.2 ± 6.01
Prox. colon NBCe1^−/−^	2.92 ± 0.67	10.72 ± 0.47	66.75 ± 6.62	77.5 ± 6.91

For PD: *P < 0.05; **P < 0.01. For *R*_t_: #*P* < 0.05, *n* = 6–8

In contrast, a significantly lower *I*_sc_ response to FSK was observed in small intestine and the cecum, but not the proximal colon of NBCe1 KO mice, (Fig. 5a-f, Table 2), suggesting a reduced Cl^−^ secretory response (because HCO_3_^−^ secretory response was not compromised, see above). Basal resistance (R) was not different between WT and KO tissue, but the FSK-induced decrease in R (presumably due to narrowing of the lateral spaces during stimulation of fluid secretion [17, 19] was significantly lower in the NBCe1 KO than WT jejunum, indicating a compromised fluid secretory response to FSK in the absence of NBCe1 (Table 2). CFTR mRNA expression was not significantly different in the studied segments (Fig. 5i).

**Fig. 6:**
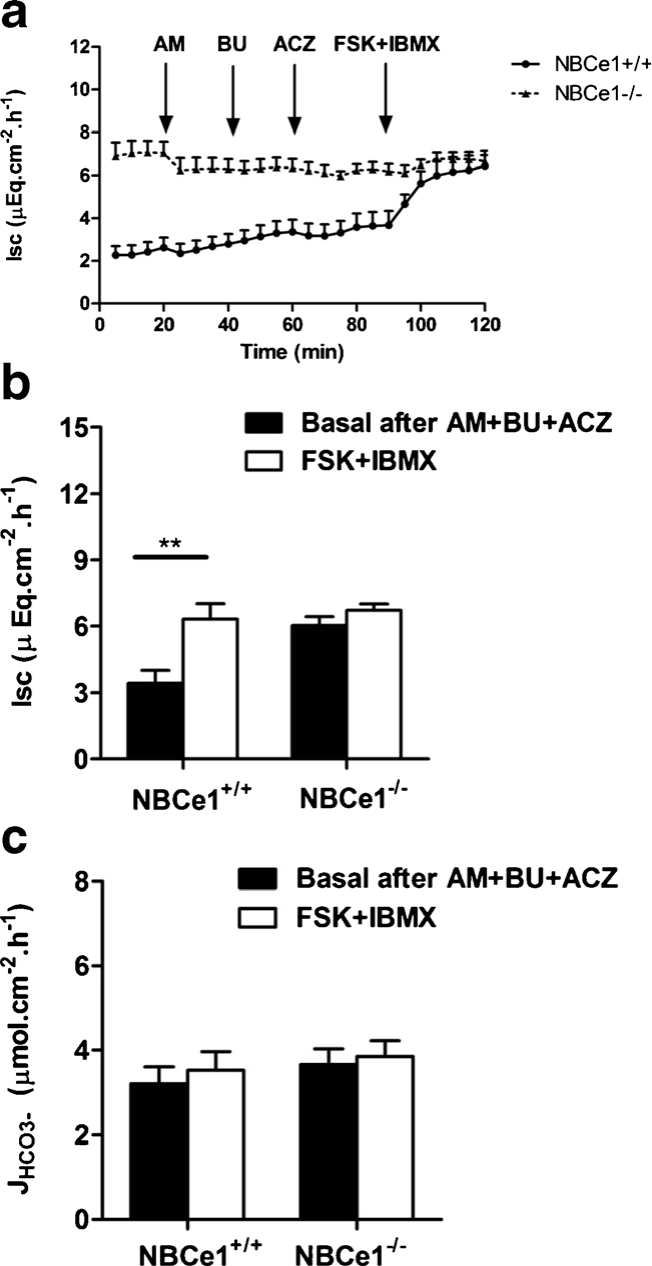
A significant FSK-induced *I*_sc_ response after NKCC1, ENaC and CA inhibition only seen in WT, not in NBCe1 KO cecal mucosa. **a**, **b** After application of luminal amiloride (10^−5^M), serosal bumetanide (10^−4^M), and bilateral acetazolamide (10^−4^M), FSK elicited a residual *I*_sc_ response in NBCe WT but not KO cecum. **c** Since no concomitant *J*_HCO3_^−^ response was observed, the *I*_sc_ response is indicative of electrogenic NBCe1-dependent Cl^−^ secretion in the WT cecum (*n* = 9)

Although the above experiments already suggested that the NBCe1 isoform may serve the purpose of a HCO_3_^−^ uptake mechanism that eventually augmented the process of Cl^−^ secretion, we further investigated this issue by sequentially inhibiting ENaC (which is upregulated in the NBCe1 KO colon ([16], ESM Fig. 3) by luminal amiloride, NKCC1 by basolateral bumetanide, and CO_2_ hydration by acetazolamide (Fig. 6a). When we applied FSK to stimulate electrogenic anion secretion under these circumstances, this resulted in a significant *I*_sc_ response without concomitant increase in the HCO_3_^−^ secretory rate in the WT but not KO cecum (Fig. 6a, b). This demonstrates that NBCe1 indeed may serve as a basolateral anion uptake mechanism for facilitating Cl^−^ secretion.

A curious observation was the effect of acetazolamide on the short circuit current. While it did not result in a significant difference on the basal *I*_sc_, acetazolamide reduced the WT *I*_sc_ response to FSK to that of the KO mucosa (Table 3). These results suggests that while the effect of carbonic anhydrase inhibition on HCO_3_^−^ secretion is via the alternative route for HCO_3_^−^ supply, namely intracellular CO_2_ hydration, the effect of carbonic anhydrase inhibition on the *I*_sc_ is via the NBCe1 itself, at least in jejunum and cecum. This suggests that NBCe1 transport is strongly dependent on extracellular (membrane bound) carbonic anhydrases in these intestinal segments.

**Table 3:** Basal and FSK-induced *I*_sc_ without inhibitor and with acetazolamide (10^−4^ M bilaterally, 10 min prior to FSK) pretreatment in NBCe1 WT and KO intestinal segments

	FSK-induced △*I*_sc_ without inhibitor (μEq cm^−2^ h^−1^)	FSK-induced △*I*_sc_ in the presence of acetazolamide (μEq cm^−2^ h^−1^)
Duodenum NBCe1^+/+^	5.65 ± 0.24	4.58 ± 0.59
Duodenum NBCe1^−/−^	3.17 ± 0.46^*^&	5.51 ± 0.92
Jejunum NBCe1^+/+^	6.92 ± 0.60	5.40 ± 0.39
Jejunum NBCe1^−/−^	3.69 ± 0.52^*^&	5.14 ± 0.85
Cecum NBCe1^+/+^	9.90 ± 0.86	3,33 ± 0.57
Cecum NBCe1^−/−^	5.31 ± 1.09^*^	2.55 ± 0.46
Prox.Colon NBCe1^+/+^	3.05 ± 0.33^*^	Not done
Prox.Colon NBCe1^−/−^	4.52 ± 0.19	

*P < 0.05, comparison between NBCe1 WT and KO mice; ^%^ *P* < 0.05, comparison in NBCe1 WT mice between the absence and presence of acetazolamide; &*P* < 0.05, comparison of FSK-induced △*I*_sc_ in NBCe1 KO mice between the absence and presence of acetazolamide. *n* = 6-8

### NBCe1 augments dipeptide-mediated acidification of the jejunal villous enterocytes

**Fig. 7:**
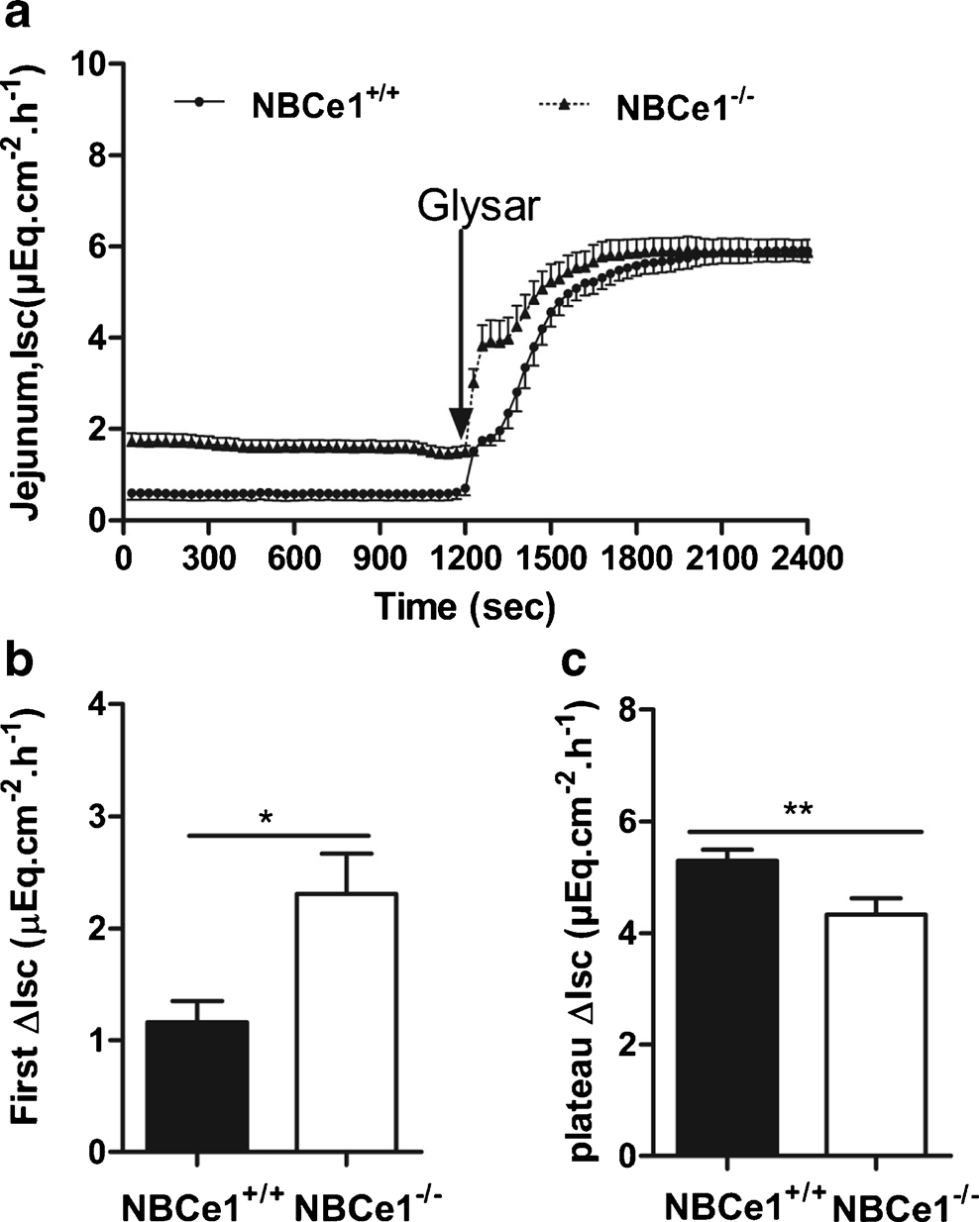
*I*_sc_ changes in response to luminal addition of GlySar in NBCe1 KO and WT jejunum. **a** *I*_sc_ was significantly higher in NBCe1 KO than WT jejunum, also under voltage clamp conditions, in the absence of any ion gradients; 20 mM GlySar caused a biphasic *I*_sc_ response. **b** The initial rapid increase in *I*_sc_ was significantly higher in the NBCe1 KO jejunum, indicating that the electrogenic influx of base via the basolateral membrane lowered the *I*_sc_ response due to electrogenic H^+^/dipeptide influx via the apical membrane. **c** The *I*_sc_ plateau, however, was significantly reduced in NBCe1 KO jejunum. *P < 0.05, *n* = 7 KO, 9 WT

Dipeptide absorption is associated with villous enterocyte acidification [11, 41]. We therefore investigated whether NBCe1 activity may be involved in PEPT1-mediated H^+^/dipeptide absorption. GlySar-mediated *I*_sc_ response, which is a function of PEPT1-mediated electrogenic H^+^/dipeptide import [11] was assessed under voltage-clamp conditions with equal ion concentrations in the luminal and basolateral bath. Even under these conditions (no HCO_3_^−^ gradient into the lumen), the basal *I*_sc_ was higher in the NBCe1 KO tissue. 20 mM GlySar in the luminal bath resulted in a biphasic increase in *I*_sc_ (Fig. 7a). The first phase of *I*_sc_ response was significantly higher in the NBCe1 KO jejunum, whereas the plateau was significantly lower (Fig. 7b). We assume that NBCe1 is being activated during the electrogenic H^+^/dipeptide absorption, because this causes both a membrane depolarization as well as an acidification of the enterocyte cytoplasm, both of which will facilitate NBCe1-mediated electrogenic HCO_3_^−^ absorption. The influx of negative charge via NBCe1 will reduce the initial GlySar-induced *I*_sc_ increase in the WT jejunum, and the pH_i_ regulatory effect of NBCe1-mediated HCO_3_^−^ absorption helps maintain membrane negativity and persistent GlySar absorption from the lumen in the WT tissue. The lower GlySar-induced plateau in NBCe1 KO jejunum indicates that NBCe1 is operative in the maintenance of pH_i_ and membrane potential during peptide absorption.

### mRNA expression of alternative acid/base transporters in the NBCe1 knockout intestine

**Fig. 8:**
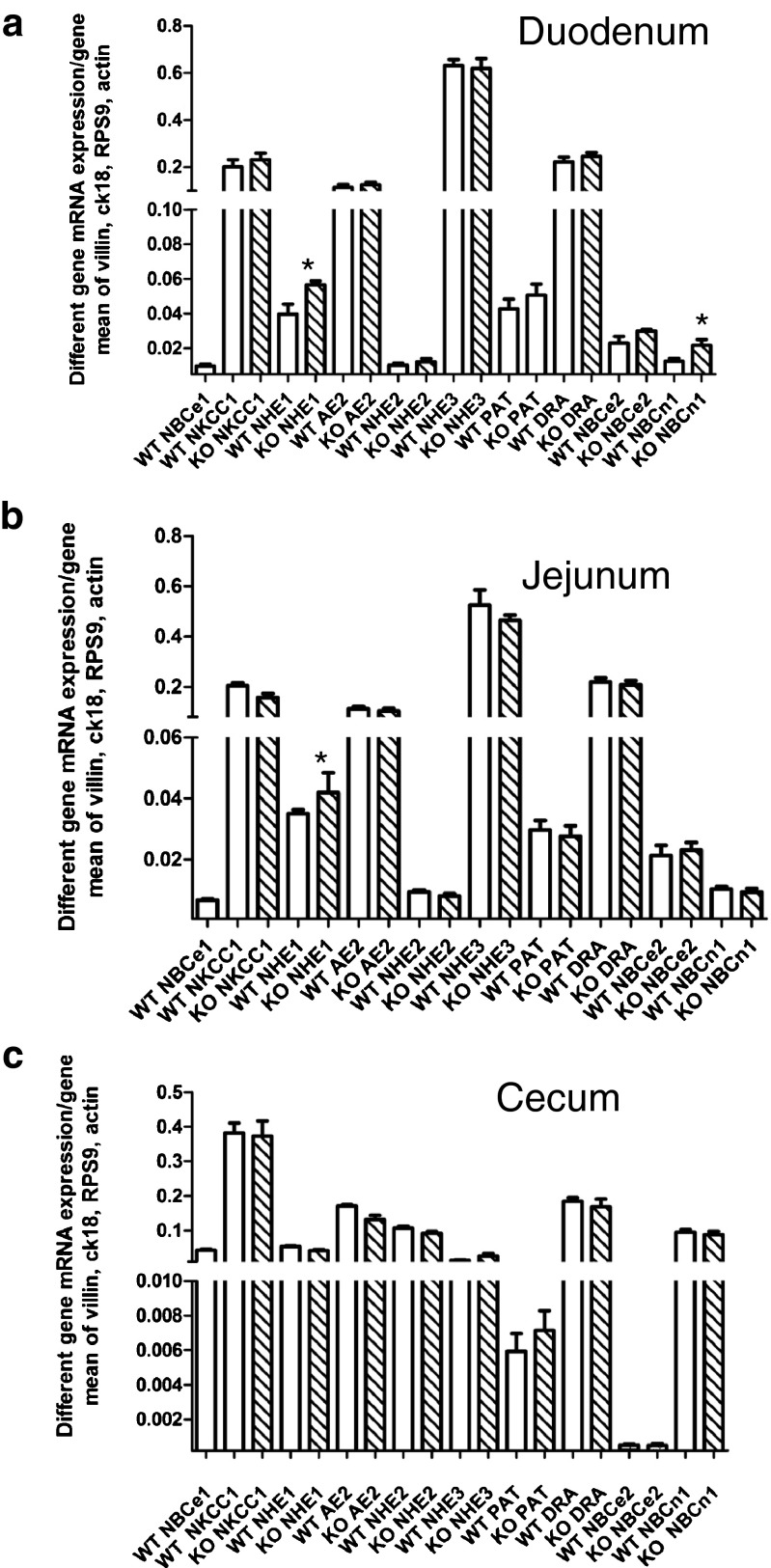
mRNA expression levels for multiple acid/base transporters in the different segments of NBCe1 KO and WT mucosa at day 13–16 days of birth. **a**–**c** mRNA expression levels for acid/base transporters were calculated relative to the geometric mean of villin, cytokeratin 18, actin and RPS9. While the mRNA expression for the major transporters for electrolyte transport (NHE3, DRA, NKCC1, AE2, CFTR) was not different between WT and KO, other pH_i_ regulators such as NHE1 and NBCn1 were significantly upregulated in the duodenum. *P < 0.05, *n* = 6

**Fig. 9:**
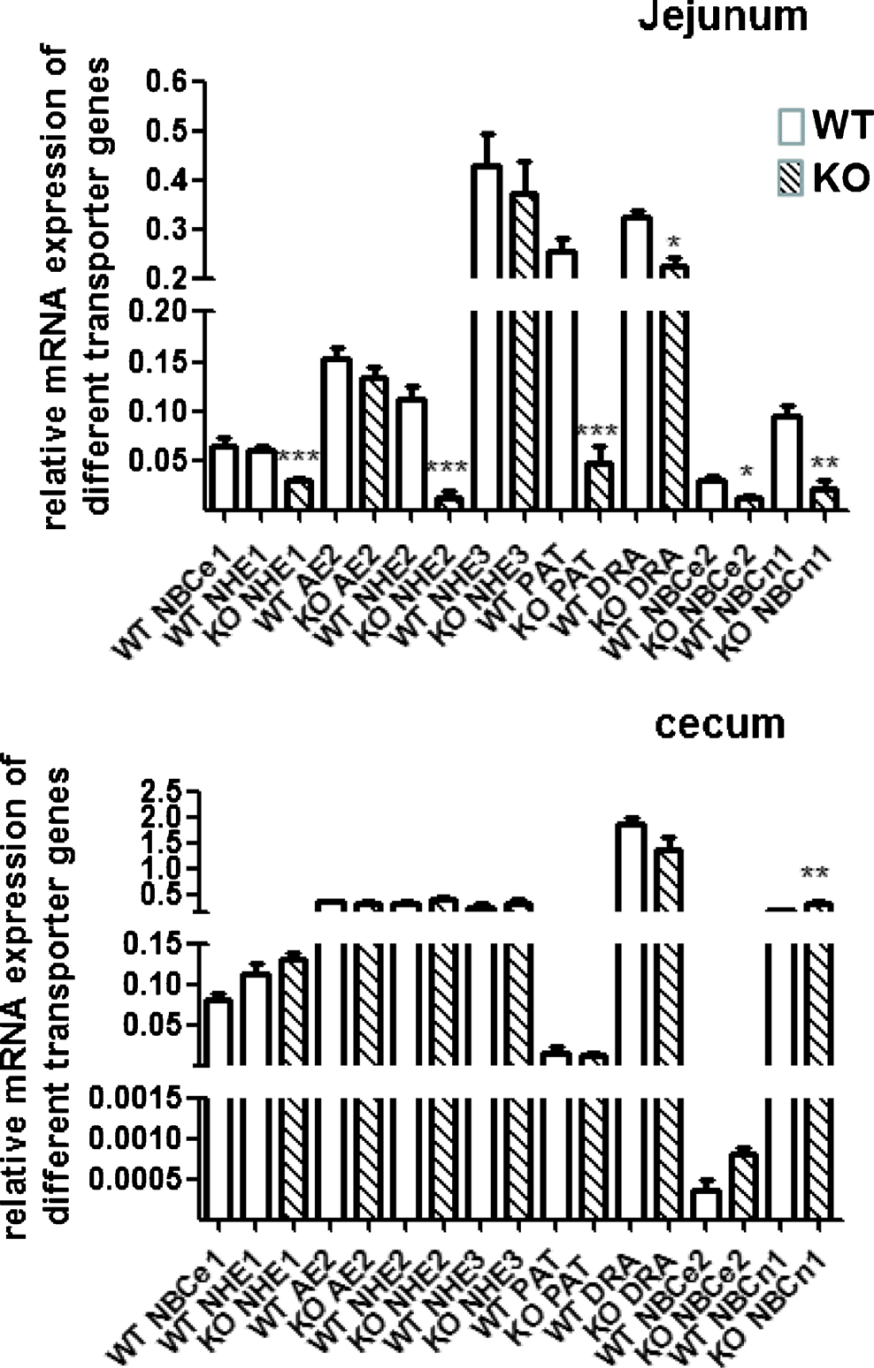
mRNA expression levels for multiple acid/base transporters in the different segments of NBCe1 KO and WT mucosa at day 18–21 days of birth, In contrast to the small differences in transporter expression levels between WT and KO intestine at 13–16 days of age, the situation changes dramatically at 18–21 days of age. The expression of a set of acid/base transporters (NHE1, NHE2, PAT1, NBCn1) was strongly decreased in NBCe1 KO compared to age-matched WT pups, selectively in the jejunum (see ESM Fig. 2 for duodenum). Upon comparison with Fig. 8, it becomes evident that the above named transporters increase their jejunal mRNA expression levels in relationship to the other measured transporters in the WT, and that this increase does not occur in the KO jejunum. *P < 0.05; ***P < 0.001, *n* = 6

The NBCe1 KO mouse is probably the best in vivo model to study adaptive changes in gene expression to chronic metabolic acidosis. We analyzed acid base status in ~14-day-old mice, when a difference in weight started to become obvious (Table 4). The mice were acidotic, but they compensated by hyperventilation. At day 13–16 after birth, the expression of most intestinal acid/base transporters was not significantly different between KO and WT mice. This suggests that our observed differences in transport functions are predominantly due to the lack of NBCe1 function itself, and that a functional compensation by other transport processes occurs rather than changes in the expression levels. Notable exceptions were an upregulation of NHE1 in duodenum and jejunum, and of NBCn1 in duodenum (Fig. 8a-c). We also analyzed acid base status and acid/base transporter mRNA expression levels at day 18–21, when the mice had actually lost weight rather than stopped gaining weight (Table 4). These mice were as severely acidotic as described by Gawenis et al. [16], and their jejunum had a brownish hue. The intestinal contents were often curd-like, but intestinal obstructions, as seen in the CFTR KO mice, are not a feature of the NBCe1 intestine. Concomitantly, the mRNA expression of a number of acid/base transporter genes were dramatically downregulated in the jejunum, while they were not affected or upregulated in duodenum and cecum (Fig. 9, ESM Fig. 2).

**Table 4:** Weight and blood gas analysis in 13–16- and 18–21-day-old NBCe1 WT and KO mice

	NBCe1^+/+^ (14–15 days)	NBCe1^+/+^ (18–20 days)	NBCe1^−/−^ (12–14 days)	NBCe1^−/−^ (18–20 days)
Body weight (g)	8.85 ± 0.66	8.92 ± 0.17	6.36 ± 0.56*	4.90 ± 0.29****^§^
pH	7.42 ± 0.01	7.41 ± 0.01	7.11 ± 0.03****	6.71 ± 0.06****^§§§§^
pCO_2_ (mmHg)	39.8 ± 4.7	34.5 ± 1.5	25.8 ± 3.6*	19.9 ± 3.1***
pO_2_ (mmHg)	267.4 ± 15.6	265.1 ± 10.9	254.1 ± 23.2	287.8 ± 6.9
sO_2_ (%)	100 ± 0.0	99.5 ± 0.1	99.7 ± 0.1	99.3 ± 0.2
HCO_3_^−^ (mmol/L)	25.1 ± 2.7	21.2 ± 0.9	7.8 ± 1.2****	2.5 ± 0.5****^§§§^
ABE (mmol/L)	1.4 ± 2.3	−2.3 ± 1.0	−21.6 ± 1.7****	
SBE (mmol/L)	1.2 ± 2.7	−2.8 ± 1.1	−19.9 ± 1.5****	−27.6 ± 1.2****^§§§^
SBC (mmol/L)	25.8 ± 2.1	22.5 ± 0.8	9.9 ± 0.9****	

*Compared between WT and KO group, §compared between 12–14 and 18–20 days in KO group, 14–16 days *n* = 7–10, 18–20 days *n* = 13–15. There were no ABE and SBC values in 18–20-days KO group because of serious metabolic acidosis. The high pO_2_ is due to the isoflurane/O_2_ mixture used for narcosis prior to taking blood

^****§§§§^ *p* < 0.0001

## Discussion

Despite the low systemic HCO_3_^−^ concentration, the gastrointestinal tract of NBCe1 knockout mice develops normally during embryogenesis and in the short lifespan of the mice (ESM Fig. 1). Therefore, the functional defects in electrolyte transport processes due to lack of NBCe1 expression can be assessed in isolated epithelial preparations from NBCe1 KO and WT mice, in which the influence of the low systemic pH and HCO_3_^−^ concentrations of NBCe1-deficient mice on pH_i_ regulation and electrolyte transport are abolished.

### Participation of NBCe1 in pH_i_ control

After NHE inhibition, a significantly lower base influx rate after an intracellular acid load was observed in jejunal villous NBCe1 KO than WT enterocytes; it was also lower in surface/cryptal mouth NBCe1 KO than in WT colonocytes, whereas no such difference was observed in NBCe1 KO and WT villous duodenocytes and in colonic crypt cells. This is consistent with the higher expression of NBCe1 than NBCn1 in the jejunal villi and the colonic surface cells. Conversely, a significant difference in base influx rate had been previously described in NBCn1 KO and WT duodenal enterocytes, and was also found in this study in the cryptal colonocytes of the proximal and mid colon, again corresponding with the zones of highest expression of this transporter [10, 38]. In addition, NBCe1 KO duodenum displayed an upregulation of both NHE1 and NBCn1 mRNA expression, which is likely to contribute to a compensated pH_i_ recovery after NHE inhibition. Steady-state pH_i_ was not significantly different between WT and KO enterocytes in most studied intestinal segments when measured fluorometrically in microdissected duodenal and jejunal villi, but this may be different in the physiological context with different ion gradients between the basolateral and the apical enterocyte compartment.

### Involvement of NBCe1 in HCO_3_^−^ secretion

Both the basal as well as agonist-stimulated HCO_3_^−^ secretory rates were not significantly different in NBCe1 KO and WT duodenal mucosa, even in the presence of ion gradients strongly favoring HCO_3_^−^ secretion, suggesting that other HCO_3_^−^ uptake mechanisms, such as NBCn1 and carbonic anhydrase-facilitated CO_2_ hydration in conjunction with NHE activity, either are more important or can compensate for the lack of NBCe1. Indeed, carbonic anhydrase inhibition decreased basal HCO_3_^−^ secretion (Fig. 4a), and NBCn1 ablation decreased FSK-stimulated HCO_3_^−^ secretion in isolated murine duodenal mucosa [10],

In the NBCe1 KO jejunum, where NBCe1 is expressed more strongly than NBCn1, a reduction in basal *J*_HCO3_^−^ was observed. Curiously, FSK-induced HCO_3_^−^ secretory response was not different from WT, even after carbonic anhydrase inhibition. In contrast to adult mice [10], NBCn1 mRNA is expressed at similar levels than NBCe1 in the jejunum of these young mice, and likely serves as an alternative pathway for HCO_3_^−^ uptake in both NBCe1 KO and WT jejunum. Of interest in this respect is also the relatively high NBCe2 expression in these suckling mice when compared to adult mouse jejunum [10]. NBCe2 displays a stoichiometry of 3:1 HCO_3_^−^ to Na^+^ and should therefore operate as a HCO_3_^−^ efflux pathway via the basolateral membrane, resulting in bicarbonate absorption in vivo, in conjunction with proton extrusion into the lumen by apical NHE3 activity. This process is inhibited by FSK, and results in an increase in HCO_3_^−^ “secretion” in an electroneutral NHE3-dependent fashion [38, 46]. This may theoretically also explain why FSK results in a similar increase in luminal alkalinisation in NBCe1 KO and WT jejunum, despite a lower basal rate in the KO jejunum.

In both WT and NBCe1 KO cecum, luminal alkalinisation rates *J*_HCO3_^−^ were very high and strongly luminal Cl^−^-dependent (Fig. 3). The likely explanation for the high cecal alkalinisation rates is that, in contrast to the duodenum and jejunum, the expression of the luminal Cl^−^/HCO_3_^−^ exchanger DRA is markedly higher than that of the luminal Na^+^/H^+^ exchanger NHE3 (Fig. 8, and [40]). The higher alkalinisation rates in NBCe1 KO than WT cecum were abolished by luminal Cl^−^ removal, suggesting that cecal enterocytes take up more Cl^−^ via the luminal Cl^−^/HCO_3_^−^ exchanger DRA when basolateral Cl^−^ uptake via coupling of NBCe1 and AE2 is absent and NKCC1 function is compromised (see below). The very strong decrease in luminal alkalinization in NBCe1 KO but not WT cecum upon CA inhibition demonstrates that NBCe1 can supply large amounts of HCO_3_^−^ for colonic Cl^−^ absorption and luminal alkalinisation, but that, on the other hand, high CA activity and basolateral acid extrusion may completely substitute for a defective NBCe1.

### Involvement of NBCe1 in intestinal Cl^−^ secretion

The measurement of intestinal *I*_sc_ is considered a good approximation of intestinal Cl^−^ secretion if certain precautions are taken into consideration: (a) electrogenic Na^+^ absorption is either inhibited or not present (as evidenced by a lack of *I*_sc_ changes after amiloride addition); (b) luminal HCO_3_^−^ output is simultaneously titrated and therefore can be taken into the calculation; (c) agonist-induced *I*_sc_ response, not the basal *I*_sc_, is taken into consideration [13]. In the NBCe1-deficient intestinal mucosa, FSK-stimulated ∆*I*_sc_ response was significantly lower in duodenal, jejunal, and cecal , but not in the proximal colonic mucosa of when compared to WT mice. Since ENaC was inhibited (cecum and colon) or not present (duodenum and jejunum) and FSK-induced HCO_3_^−^ output was not different, the difference in FSK-induced *I*_sc_ response reflects a difference in electrogenic Cl^−^ secretion. In the jejunum, the strong difference in the increase in epithelial ΔR_t_, which is an indicator of the CFTR activation-dependent lateral space collapse due to fluid secretion in the jejunum [17], is a good indicator of a strong reduction of FSK-induced fluid secretion (Table 2). Therefore, the data suggest that jejunal anion and fluid secretion is severely compromised in the absence of NBCe1 in these young mice.

Since this fluid secretory defect previously escaped notice, it was speculated that the small intestinal impactions noticed in the NBCe1-deficient or defective mice [16, 30] may be due to the hyperaldosteronism-induced upregulation of NHE3, thus leading to increased absorption. But NHE3 mRNA expression levels were not upregulated in the jejunum of NBCe1 KO compared to WT mice (Fig. 8), a finding that is in agreement with our previous observation that hyperaldosteronism induced by low-salt diet (Riederer, unpublished observations) or by Slc26a3 deficiency [44] had a strong effect on colonic, but not on jejunal NHE3 expression levels. Although an increase in ENaC-mediated Na^+^ absorption occurs in the colon of NBCe1 KO mice ([16], ESM Fig. 3), the impactions were described in the small intestine. The severe fluid secretory defect per se may be sufficient to explain intestinal impactions.

The involvement of NBCe1 in Cl^−^ (as opposed to HCO_3_^−^) secretion was also studied in the cecal mucosa (Fig. 6). After the inhibition of basolateral Cl^−^ uptake by NKCC1 (by serosal bumetanide) and carbonic anhydrase-facilitated intracellular HCO_3_^−^ generation by acetazolamide, FSK still elicited a significant ∆*I*_sc_ in WT, but not in NBCe1 KO cecum. Since no simultaneous change in *J*_HCO3_^−^ was recorded, the ∆*I*_sc_ is due to Cl^−^ secretion. This experiment demonstrates that NBCe1 is able to sustain Cl^−^ secretion, most likely via coupling with basolateral AE2, as suggested by Walker et al. [42]. This mechanism is operative even after complete carbonic anhydrase inhibition.

### Influence of NBCe1 activity on basal *I*_sc_

In duodenal, jejunal and cecal, but not in proximal colonic mucosa, basal *I*_sc_ was significantly higher in the NBCe1 KO than WT mucosa, both under open-circuit conditions and voltage clamp conditions (Fig. 5). This higher *I*_sc_ was not abolished by luminal amiloride or basolateral bumetanide (although our NBCe1 KO mice also were hyperaldosteronemic, as reported by Gawenis et al. [16], and an increased ENaC expression (ESM Fig. 3) as well as activity (data not shown) was noted in colonic mucosa. We therefore believe that the most straightforward explanation is that the lower *I*_sc_ in the WT mice may be due to the electrogenic influx of HCO_3_^−^ across the basolateral membrane of enterocytes (occurring in cells that are also operative in Cl^−^ and/or HCO_3_^−^ export via the apical membrane via CFTR, DRA, and/or PAT-1). CFTR channels are expressed at relative high levels in the villous epithelium in the duodenum and to some extent also in the jejunum, but are strongly crypt-expressed in the cecum and colon, whereas NBCe1 displays a surface cell-predominant colonic expression [23]. DRA and PAT1 expression is surface-predominant, thus occurring in the same cells that express NBCe1 in the basolateral membrane [22, 36, 40, 43, 44]. At neutral pH and in the absence of nutrients, CO_2_ or secretagogues in the luminal bath (i.e., the conditions of the Ussing-chamber experiments), the major acid loading mechanism for the surface/villous is apical Cl^−^HCO_3_^−^ exchange. This acid load can be counteracted by apical NHE3, but if that was the only mechanism, it would not explain high Cl^−^-dependent alkalinisation rates in several segments of the GI tract including the cecum (where NHE3 expression is low), because the overall result would be CO_2 _and water output into the lumen. In addition, it would not explain the differential expression of NHE3 and PAT-1/DRA in the different intestinal segments. Basolateral NBCe1-mediated HCO_3_^−^ import may counteract some of the DRA-mediated enterocyte acid loading, which may explain the significantly different basal *I*_sc_ between NBCe1 KO and WT small intestinal and cecal mucosa. The proximal colonic surface enterocytes are thought to exchange intracellular HCO_3_^−^ for the short chain fatty acid anions, and we assume that the reason for the high surface cell expression of NBCe1 in the surface cells of the proximal colon is related to the acid load imposed upon these cells by short-chain fatty acid absorption.

### Importance of NBCe1 in peptide absorption

Enterocyte acid loads in the small intestine are imposed during nutrient absorption, most notably during proton-coupled peptide via PepT1. Current dogma envisions peptide absorption to be driven by a proton gradient [41] and enterocyte pH_i_ homeostasis during peptide absorption mediated via the apical sodium/hydrogen exchanger NHE3 [39, 41]. However, an acidic microclimate pH of the small intestine results in an inhibition of luminal Na^+^/H^+^ exchange by competition of the highly affinitive protons with Na^+^ at the external binding site. The luminally expressed Slc26 anion transporter family member Slc26a6 (PAT-1) is able to import base during peptide-induced acidification [37], but would also be inhibited at low luminal pH (due to lack of base equivalents). In contrast, NBCe1 activity is activated both by the low pH_i_ and by the membrane depolarization caused by proton-coupled dipeptide influx (Fig. 7a), and the difference in the *I*_sc_ plateau phase after GlySar application demonstrates that peptide absorption is indeed lower in the NBCe1 KO mouse (Fig. 7c). The initial *I*_sc_ response to GlySar was faster in the KO (Fig. 7b), indicating that an activation of NBCe1-mediated electrogenic base intake via the basolateral side, stimulated by PepT-1-mediated intracellular acid load [9], results in a lumen-positive current (and slows the increase in lumen-negative current by apical proton influx). The difference in maximal GlySar-induced *I*_sc_ between WT and KO became larger when the luminal pH was lowered (data not shown), suggesting that under physiological conditions NBCe1 is a crucial homeostatic mechanism to ensure adequate peptide absorption. Because most modes of nutrient absorption acidify the enterocytes and/or depolarize the membrane potential, NBCe1 may be activated during all modes of nutrient absorption.

### Response of the intestinal mucosa to metabolic acidosis

NBCe1 KO intestine develops normally during the first weeks of life despite the severe metabolic acidosis. To our surprise, the mRNA expression for the major electrolyte transporters such as NHE3, AE2, NKCC1, DRA, etc. was not different between KO and WT, In contrast, the base uptake mechanisms NBCn1 and NHE1 mNRNA expression was significantly upregulated in duodenal mucosa, NHE1 in jejunal mucosa, at two weeks after birth, and NBCn1 and NBCe2 at 3 weeks after birth in the cecum and duodenum. This suggests that expression of these base uptake mechanisms is upregulated by systemic acidosis, possibly as a compensatory mechanism.

### Jejunal failure with severe weight loss and progressive acidosis develops at the end of life in NBCe1 KO mice

The NBCe1 mice not only failed to increase weight but actually lost weight in the third week of life. This was accompanied by sudden development of large differences in the mRNA expression of a variety of acid/base transporters between WT and KO pups, most strikingly for NHE2 and Slc26a6 (PAT1), selectively in jejunal tissue (Fig. 9, ESM Fig. 2). Concurrently, the metabolic acidosis worsened dramatically, suggesting that the increased energy demands of the mice due to the severe hyperventilation and the reduced jejunal digestive function results in jejunal organ failure, progressive malnutrition, worsening acidosis and death.

## Electronic supplementary material

Below is the link to the electronic supplementary material.

Supplementary Table 1 (DOCX 17 kb)

Supplementary Table 2 (DOCX 17 kb)

Supplementary Fig. 1 (DOCX 2390 kb)

Supplementary Fig. 2 (DOCX 20 kb)

Supplementary Fig. 3 (DOCX 189 kb)

## Abbreviations

ACZ: Acetazolamide
AM: Amiloride
BU: Bumetanide
cAMP: Cyclic adenosine monophosphate
DIDS: 4,4′-Diisothiocyanatostilbene-2,2′-disulfonic acid disodium salt hydrate
FSK: Forskolin
NBCe1: The electrogenic Na^+^HCO_3_^−^ cotransporter
NBCn1: The electroneutral Na^+^:HCO_3_^−^ cotransporter
*I*_sc_: Short-circuit current
pH_i_: Intracellular pH
TTX: Tetrodotoxin
ENaC: Epithelial sodium channel
CK18: Cytokeratin 18
RPS9: Ribosomal protein S9
