# Supplementary material for: Defective small intestinal anion secretion, dipeptide absorption, and intestinal failure in suckling NBCe1-deficient mice
Source: Pflugers Arch. 2016 May 26;468:1419–32. doi: 10.1007/s00424-016-1836-3 (PMC4951514; doi:10.1007/s00424-016-1836-3)
Supplement: Supplementary file 1 — (DOCX 17 kb) [file 424_2016_1836_MOESM1_ESM.docx]

**Supplementary Tables and Figures**

**Supplementary Table 1**

Buffer compositions for Ussing Chamber experiment (in mM)

| **Buffer** | **A** | | **B** | | **C** | |
| --- | --- | --- | --- | --- | --- | --- |
|  | **Basolateral** | **Apical** | **Basolateral** | **Apical** | **Basolateral** | **Apical** |
| **NaCl** | **108** | **154** | **108** | **108** | **108** | **154** |
| **KCl** | **3** | **/** | **3** | **3** | **3** | **/** |
| **MgSO_4_.7H_2_O** | **1.3** | **/** | **1.3** | **1.3** | **1.3** | **/** |
| **KH_2_PO_4_** | **2.25** | **/** | **2.25** | **2.25** | **2.25** | **/** |
| **NaHCO_3_** | **22** | **/** | **22** | **22** | **22** | **/** |
| **CaCl_2_.2H_2_O** | **2** | **/** | **2** | **2** | **2** | **/** |
| **D-Glucose** | **8.9** | **/** | **10** | **/** | **8.9** | **/** |
| **TTX** | **10^-3^** | **/** | **10^-3^** | **/** | **10^-3^** | **/** |
| **Na-pyuvat** | **10** | **/** | **/** | **/** | **10** | **/** |
| **Indomethacin** | **3*10^-2^** | **/** | **3*10^-2^** | **/** | **3*10^-2^** | **/** |
| **HEPES** | **/** | **/** | **10** | **10** | **/** | **/** |
| **Mannitol** | **/** | **/** | **^&^20** | **10** | **/** | **/** |
| **Glysar** | **/** | **/** | **/** | **^&^20** | **/** | **/** |
| **Na^+^-gluconate** | **/** | **/** | **/** | **/** | **/** | **^#^154** |

A: In normal experiment, basolateral solution was gassed with 95%O_2_ and 5%CO_2_, and apical solution was gassed with 100% O_2_.

B. In the Glysar experiment, & means after 30 min stable baseline, Glysar was performed and identical osmolarities were achieved by adjustment with mannitol. Both basolateral and luminal solution were gassed with 95%O2 and 5%CO2.

C. In the Cl^-^ free experiment, # means after 30min stable baseline, Na^+^-gluconate was performed to replace of NaCl in the luminal bath and gassed with 100% O2.
