# Supplementary material for: Defective small intestinal anion secretion, dipeptide absorption, and intestinal failure in suckling NBCe1-deficient mice
Source: Pflugers Arch. 2016 May 26;468:1419–32. doi: 10.1007/s00424-016-1836-3 (PMC4951514; doi:10.1007/s00424-016-1836-3)
Supplement: Supplementary file 2 — (DOCX 17 kb) [file 424_2016_1836_MOESM2_ESM.docx]

**Supplementary Table 2**

Buffer compositions for the pHi measurements (in mM)

| **Buffer** | **A** | **B** | **C** | **D** | **E** |
| --- | --- | --- | --- | --- | --- |
| **Hepes** | **10** | **10** | **10** | **10** | **10** |
| **Tris** | **5** | **5** | **5** |  |  |
| **KH_2_PO_4_** | **2.25** | **2.25** | **2.25** | **2.25** | **2.25** |
| **K_2_HPO_4_** | **1.5** | **1.5** | **1.5** | **1.5** | **1.5** |
| **MgSO_4_** | **1.2** | **1.2** | **1.2** | **1.2** | **1.2** |
| **Ca^2+^-gluconate** | **1.2** | **1.2** | **1.2** | **1.2** | **1.2** |
| **Na^+^-gluconate** | **98** | **56** |  | **20** |  |
| **Glucose** | **10** | **10** | **10** |  |  |
| **(NH_4_)_2_SO_4_** |  | **27** |  |  |  |
| **TMA-gluconate** |  |  | **96** |  |  |
| **K-gluconate** |  |  |  | **118** | **118** |
| **NaHCO_3_** | **22** | **24** |  | **2** | **22** |
| **CholineHCO_3_** |  |  | **24** |  |  |

A: The villi or crypts were perfused with prewarmed Cl^-^-free basal solution gassed with carbogen (5%CO_2_-95%O_2_) for 20min for a stable baseline reading.

B. The villi or crypts were acidified using an ammonium prepulse (27mM (NH_4_)_2_SO_4_ isotonically replacing Na^+^-Gluconate) gassed with carbogen for 5 min.

C. The villi or crypts were then perfused with Na^+^-free buffer (TMAGluconate isotonically replacing Na^+^-Gluconate and CholineHCO_3_ isotonically replacing NaHCO_3_ ) gassed with carbogen until pH_i_ reached its lowest value plateu. Subsequently, 50μM Hoe642 and 20μM S1611 were added to the Na^+^-free buffer, after 2-3 min, the buffer was switched to Na^+^-containing buffer, also containing 50μM Hoe642 and 20μM S1611 gassed with carbogen.

D. Calibration Cl^-^-free buffer, pH 6.6 gassed with carbogen.

E. Calibration Cl^-^-free buffer, pH 7.4 gassed with carbogen.
