# Supplementary material for: Defective small intestinal anion secretion, dipeptide absorption, and intestinal failure in suckling NBCe1-deficient mice
Source: Pflugers Arch. 2016 May 26;468:1419–32. doi: 10.1007/s00424-016-1836-3 (PMC4951514; doi:10.1007/s00424-016-1836-3)
Supplement: Supplementary file 3 — (DOCX 2390 kb) [file 424_2016_1836_MOESM3_ESM.docx]

**
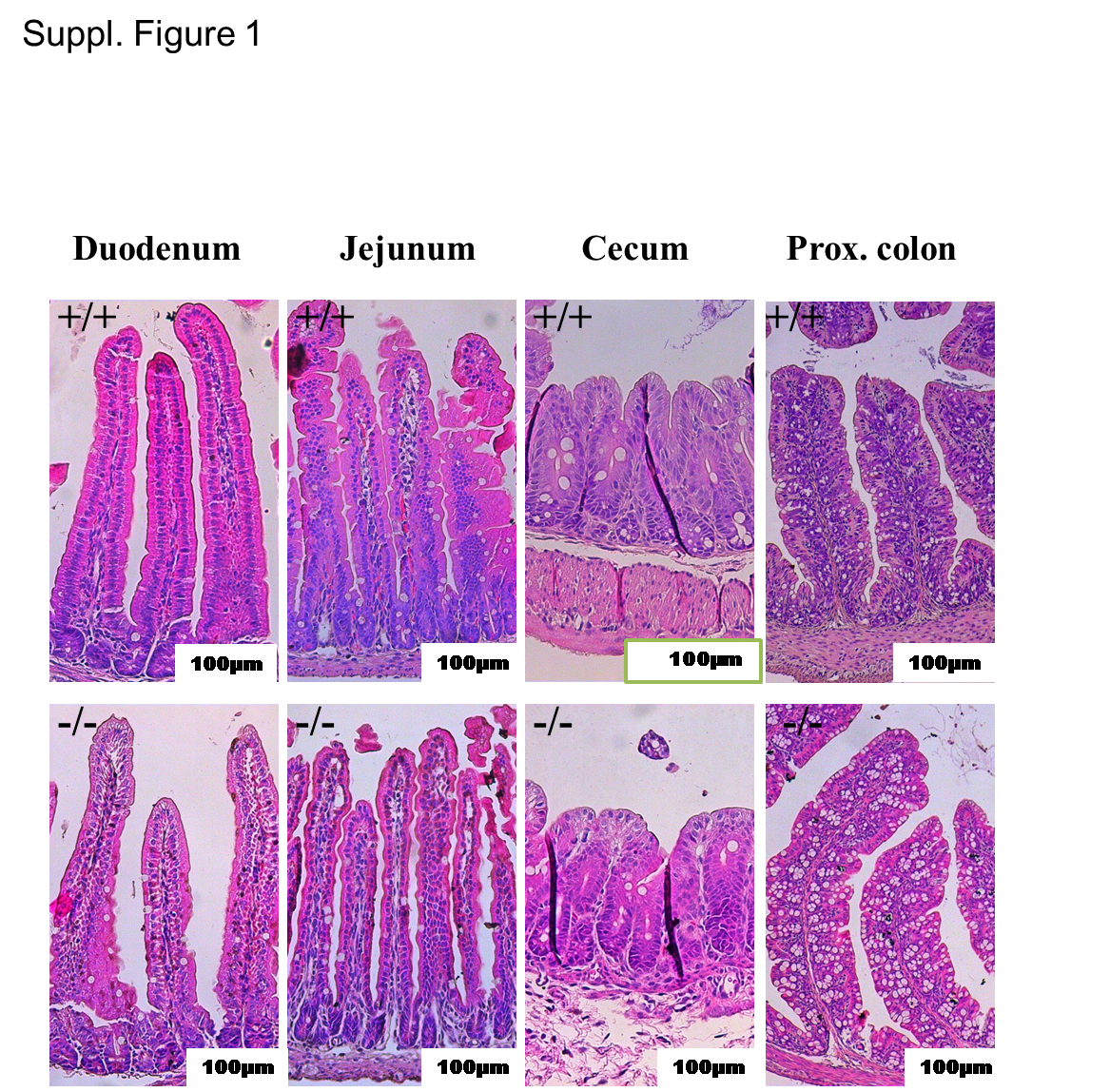
**

**Suppl. Figure1: Histology of different segments of intestine in NBCe1 WT and KO mice**

No difference were observed in the duodenum, Jejunum, Cecum and Proximal colon between NBCe1 WT and KO mice with classical H&E staining
