# Supplementary material for: Defective small intestinal anion secretion, dipeptide absorption, and intestinal failure in suckling NBCe1-deficient mice
Source: Pflugers Arch. 2016 May 26;468:1419–32. doi: 10.1007/s00424-016-1836-3 (PMC4951514; doi:10.1007/s00424-016-1836-3)
Supplement: Supplementary file 4 — (DOCX 20 kb) [file 424_2016_1836_MOESM4_ESM.docx]

**Suppl. Figure 2**

**
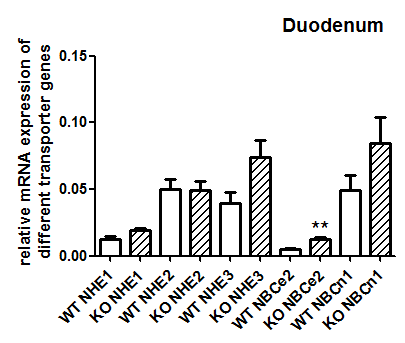
**

**Suppl. Figure 2: Transporter mRNA expression in the duodenum of 18-21 days old NBCe1 WT and KO mice**

The mRNA expression levels in the duodenum (and jejunum, not shown) were assessed with a different qPCR thermocycler for this set of experiments. The results in the jejunum (strongly depressed mRNA expression for NHE2, etc.) was similar than in Figure 9. We then repeated the experiments with different pups and the results are shown in Figure 9. The duodenum was not studied again. Reference genes were RPS9, villin, actin, CK18 and beta2microglobulin.
