# Supplementary material for: Defective small intestinal anion secretion, dipeptide absorption, and intestinal failure in suckling NBCe1-deficient mice
Source: Pflugers Arch. 2016 May 26;468:1419–32. doi: 10.1007/s00424-016-1836-3 (PMC4951514; doi:10.1007/s00424-016-1836-3)
Supplement: Supplementary file 5 — (DOCX 189 kb) [file 424_2016_1836_MOESM5_ESM.docx]

**
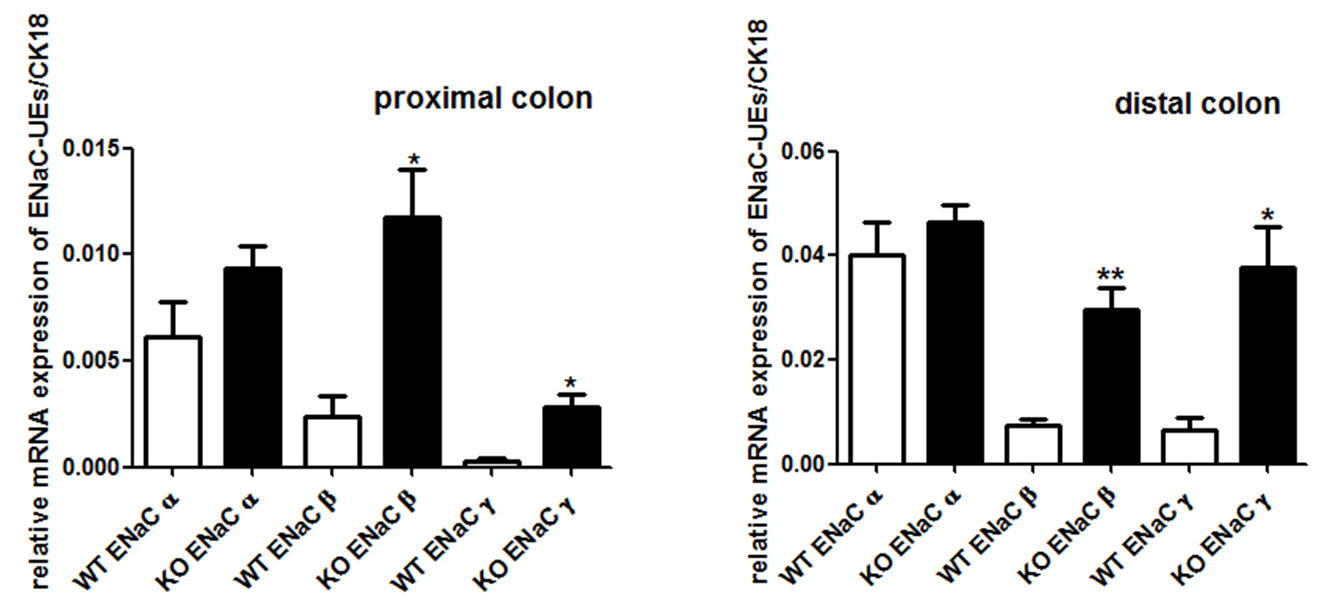
Suppl. Figure 3**

**Suppl. Figure 3: ENaC subunit expression in the proximal and distal colon of NBCe1 WT and KO mice**

In the proximal colon, ENaC subunit expression is very small in WT but markedly upregulated in KO mice, corresponding to a significant decrease in basal Isc upon luminal amiloride addition in chambered mucosa (data not shown). In the distal colon, significant ENaC expression is seen in WT, but upregulated in KO mucosa. Please not the different Y axis scale between proximal and distal colon. n= 4-5, *p<0.05, **p<0.01
